# Editorial: Cognitive Aspects of Interactive Technology Use: From Computers to Smart Objects and Autonomous Agents

**DOI:** 10.3389/fpsyg.2019.01078

**Published:** 2019-05-14

**Authors:** Amon Rapp, Maurizio Tirassa, Tom Ziemke

**Affiliations:** ^1^Computer Science Department, University of Turin, Turin, Italy; ^2^ICxT - ICT and Innovation for Society and Territory, University of Turin, Turin, Italy; ^3^Psychology Department, University of Turin, Turin, Italy; ^4^Cognition and Interaction Lab, Department of Computer and Information Science, Linköping University, Linköping, Sweden; ^5^Interaction Lab, School of Informatics, University of Skövde, Skövde, Sweden

**Keywords:** human-computer interaction, wearable technologies, virtual reality, artificial intelligence, affordances

The current advancements in many interactive technology fields, and the consequent spread of digital and intelligent devices in the consumer market, give the opportunity of deepening, exploring, and even rethinking the foundations of human technology use.

The increasing adoption of wearable and self-tracking technologies (Rapp et al., 2015), for instance, is changing how people reflect on themselves (Rapp and Tirassa, 2017), think of their past (Matassa et al., 2013; Elsden et al., 2016), perform physical activity and sport (Rapp and Tirabeni, 2018), and manage their health (Schroeder et al., 2018), encouraging us to explore the impacts of such technologies on mind and behavior. Likewise, people spend more and more time in digital environments, like video games (Rapp, 2017), social media (Lu et al., 2018), and virtual organizations (Reinecke et al., 2013), whereby these virtual and augmented realities are blurring the boundaries between the digital and the material world, potentially affecting how we experience and perceive what we call the reality. The miniaturization of sensors and the rise of the Internet of Things (Atzori et al., 2010) are further making traditional human-computer interfaces disappear (Console et al., 2013), at the same time modifying the affordances that are commonly associated to everyday objects (Rapp and Cena, 2015). Lastly, the increasing ubiquity of different types of interactive robots and autonomous agents, suggests that we investigate in-depth how we humanize artificial entities (Warshaw et al., 2015), how we socially interact with them (Rapp, 2018), and how we understand their behavior (Thellman et al., 2017).

These represent only a few examples of technological changes that are reconfiguring how we interact with “tools,” which can inspire a renewed discussion on human-technology interaction. This volume precisely explores how humans create, interact, account for, and are impacted by emerging interactive technologies. It puts together 17 high-quality works, spanning from research on social cognition in human-robot interaction to studies on neural changes triggered by Internet use, also tackling relevant technological and theoretical issues that foster us to rethink how we conceptualize technology, its use, and its development. In other words, this volume addresses relevant issues at different levels, including biological, psychological, theoretical, and technological/design levels.

As for the *biological level*, Liu et al. investigate the neural mechanisms underlying Internet search, discovering that it impacts human brain functions: their study results suggest that Internet search enhances the spatial information processing, but also may alter the memory system, making individuals less engaged in remembering information. Smyk et al. conduct a study with 20 participants investigating how sensorimotor oscillatory electroencephalogram (EEG) activity can be affected by the perceived nature of a task partner, human or robot, during a novel “reciprocal touch” paradigm. The results provide evidence for differences in attentional and tactile processing when interacting with human and robotic partners.

With reference to the *psychological level*, Hou et al. try to understand individual differences in the use of social network sites by surveying 714 Chinese students in order to assess how personality traits relate to excessive use of WeChat and Weibo. They find that neuroticism, loneliness, and external locus of control have positive correlations with excessive use of Weibo and WeChat, while agreeableness, social support, and social interaction negatively correlate to their excessive use. Further, they discover that the two social network sites fulfill different needs and thus attract people with different personality traits. Reichenberger et al. explore the potentiality of virtual environments in conducting social fear conditioning related experiments. They show that social fear can successfully be induced and extinguished using virtual reality, providing insights into learning and unlearning of social fear. Whereas, Kätsyri explores the reasons underlying the sense of eeriness and lack of familiarity that we may experience when we observe virtual characters. Results of an experiment with 64 participants, asking them to learn and recognize a set of virtual, and real faces, seem to suggest that lesser perceptual expertise may contribute to the lack of subjective familiarity with virtual faces.

Böffel and Müsseler investigate an important phenomenon in human-avatar interaction: when spatial dissociation between the user's and the avatar's orientations arises as a consequence of the task handled, the user has to adopt the avatar's perspective and identify herself with it. A study is then set to identify the conditions that benefit this change of perspective: the finding is that perceived ownership, elicited by interaction instructions leading to effector congruency between the participant's hands and the hands of the avatar, benefits perspective taking. Morganti involves 61 participants in order to understand if different embodied affordances could provide different knowledge organization during wayfinding by using distinct spatial simulations. The results show that different embodiments afforded by different environments and the increasing complexity in turn types result in different spatial outcomes.

As for the *theoretical level*, Osiurak et al. attempt to provide a structured way of organizing the literature about the cognitive processes involved in the different interactions we have with tools by proposing a theoretical framework organized into three levels. The first level describes how we interact when using physical technologies which increase our sensorimotor abilities; the second level pertains to sophisticated technologies, for which we do not systematically understand the underlying working principles; the third level tackles symbiotic technologies, which link our brain directly to machines. In doing so, they highlight the key role of technical and practical reasoning, which could be undermined by the increasing use of sophisticated and symbiotic tools. Likewise, Duus et al. use the extended mind theory to explore how human-tech hybrids, represented by humans interacting with mobile phones, smart watches, and wearable activity trackers, gain and enact collective skills, how agency is expressed and affects the interaction, and what the darker sides are of being a human-tech hybrid. The proposed concept of agency pendulum, which seen agency swinging between the human and the device depending on the situation, reflects the dynamism of agency in these hybrid entities. In his article, Baber retraces the historical roots of the concept of affordance and how it has been applied to interaction design. In reaffirming its fundamental role for understanding “interactivity,” Baber further develops the concept by extending it to the interaction with “smart objects,” which sense how they are being used, communicate with each other, and provide prompts to solicit certain actions. Here, the human-object-environment system pursues shared intentions and goals, and affordances become both the means by which actions are encouraged, and the manner in which intentions are identified and agreed.

Honig and Oron-Gilad present a literature review of 52 studies that explore when people perceive and resolve robot failures, how robots communicate failure, how failures influence people's perceptions and feelings toward robots, and how such effects can be mitigated. On the basis of this review, they develop a model of information processing for robotic failures describing how individuals perceive, process, and act on failures in human-robot interaction. Musetti and Corsano present an interesting perspective for conceptualizing Internet not as a tool, but as a social environment. As people are part of an information society and can access whatever information they lack whenever they want, no boundaries between their online and offline lives can still be traced clearly. As a consequence of this shift in theorizing, main models of Internet-related pathologies, like Internet addiction, need to be rethought so to avoid pathologizing normal behaviors. In the same vein, Veissière and Stendel, Veissière and Stendel aim to recast current understandings of the mechanisms involved in the addictive use of smartphones in a broader evolutionary focus, suggesting that it is the social expectations and rewards of connecting with other people and seeking to learn from them that yield and sustain addictive behaviors. They thus propose a hypernatural monitoring model of smartphone addiction grounded in a general social rehearsal theory of human cognition.

Finally, as for the *technological/design level*, Triberti et al. focus on the role of emotions in designing interactive technologies, highlighting that designers can not only rely on aesthetic and engagement aspects of interaction, but also on emotions as cognitive processes and active agents of interaction, in order to create innovative and effective devices. van der Kuil et al. evaluate the usability of a serious game addressed to aid patients in the development of compensatory navigation strategies after a brain injury. Results show that mouse controlled interaction in 3D environments is more effective than keyboard interaction, that patients prefer video-based instructions over text-based instructions, and that feedback timing has no effect on performance and motivation. This may provide useful insight for the design of serious games aiming to transfer skills from virtual environments to real-life situations. Lovato and Piper acknowledge the growing availability of voice interfaces, making it possible for children to ask questions via Internet search even before they have learned to read and write. Drawing on human-computer interaction research, they thus review studies of how children look for information, and of how they perceive and understand the informational and social roles of technology. This review leads to important considerations for the design of future voice-based search interfaces. Lastly, Macedonia et al. emphasize how guided embodiment is an essential feature in intelligent tutoring systems for second language learning and aphasia rehabilitation, as it increases efficiency in the learning process. To enable the system of guiding the user through embodiment, the authors suggest that the system tracks users' gestures and provide corrective feedback, so that sensor technologies are paramount. The authors thus provide an overview of the sensor technologies that can be used to this aim, ranging from camera-based systems to sensing textiles.

These 17 articles give a snapshot of the current perspectives on the foundations of human interaction with tools and technology, proposing opportunities for debating emerging issues about the design and the understanding of novel interactive devices. We hope that readers will find the articles thought-provoking and insightful, encouraging them to move the debate forward.

## Author Contributions

AR drafted this editorial. All authors provided critical comments and approved it for publication.

### Conflict of Interest Statement

The authors declare that the research was conducted in the absence of any commercial or financial relationships that could be construed as a potential conflict of interest.

## References

[B1] Atzori L. Iera A. Morabito G. (2010). The internet of things: a survey. Comp. Netw. 54, 2787–2805. 10.1016/j.comnet.2010.05.010

[B2] Console L. Antonelli F. Biamino G. Carmagnola F. Cena F. Chiabrando E. (2013). Interacting with social networks of intelligent things and people in the world of gastronomy. ACM Transac. Interact. Intellig. Syst. 3:38 10.1145/2448116.2448120

[B3] Elsden C. Kirk D. S. Durrant A. C. (2016). A quantified past: toward design for remembering with personal informatics. Hum. Comput. Interact. 31, 518–557. 10.1080/07370024.2015.1093422

[B4] Lu Z. Xia H. Heo S. Wigdor D. (2018). You watch, you give, and you engage: a study of live streaming practices in China, in Proceedings of the 2018 CHI Conference on Human Factors in Computing Systems (CHI '18) (New York, NY: ACM; Paper 466), 13.

[B5] Matassa A. Rapp A. Simeoni R. (2013). Wearable accessories for cycling: tracking memories in urban spaces,in Proceedings of the 2013 ACM Conference on Pervasive and Ubiquitous Computing Adjunct Publication (UbiComp '13 Adjunct) (New York, NY: ACM), 415–424. 10.1145/2494091.2495973

[B6] Rapp A. (2018). Social game elements in World of Warcraft: Interpersonal relations, groups and organizations for gamification design. Inter. J. Hum. Comput. Interact. 34, 759–773. 10.1080/10447318.2018.

[B7] Rapp A. Cena F. (2015). Affordances for self-tracking wearable devices, in Proceedings of International Symposium on Wearable Computers, ISWC 2015 (New York, NY: ACM), 141–142. 10.1145/2802083.2802090

[B8] Rapp A. Cena F. Kay J. Kummerfeld B. Hopfgartner F Plumbaum T. (2015). New frontiers of Quantified Self: finding new ways for engaging users in collecting and using personal data, in Adjunct Proceedings of the 2015 ACM International Joint Conference on Pervasive and Ubiquitous Computing and Proceedings of the 2015 ACM International Symposium on Wearable Computers (UbiComp/ISWC'15 Adjunct) (New York, NY: ACM), 969–972. 10.1145/2800835.2807947

[B9] Rapp A. Tirabeni L. (2018). Personal informatics for sport: meaning, body, and social relations in amateur and elite athletes. ACM Transac. Comput. Hum. Interact. 25, 1–30. 10.1145/3196829.

[B10] Rapp A. Tirassa M. (2017). Know Thyself: A theory of the self for personal informatics. Hum. Comput. Interact. 32, 335–380. 10.1080/07370024.2017.1285704

[B11] Rapp A (2017). Drawing inspiration from world of warcraft: gamification design elements for behavior change technologies. Interact. Comput. 29, 648–678. 10.1093/iwc/iwx001

[B12] Reinecke K. Nguyen M. K. Bernstein A. Näf M. Gajos K. Z. (2013). Doodle around the world: online scheduling behavior reflects cultural differences in time perception and group decision-making, in Proceedings of the 2013 Conference on Computer Supported Cooperative Work (CSCW'13), February 23–27 (San Antonio, TX), 45–54.

[B13] Schroeder J. Chung C. F. Epstein D. A. Karkar R. Parsons A. Murinova N. (2018). Examining self-tracking by people with migraine: goals, needs, and opportunities in a chronic health condition, in Proceedings of the 2018 Designing Interactive Systems Conference (DIS '18) (New York, NY: ACM), 135–148.

[B14] Thellman S. Silvervarg A. Ziemke T. (2017). Folk-psychological interpretation of human vs. humanoid robot behavior: exploring the intentional stance toward robots. Front. Psychol. 14:1962 10.3389/fpsyg.2017.01962 PMC5694477 29184519

[B15] Warshaw J. Matthews T. Whittaker S. Kau C. Bengualid M. Smith B. A. (2015). Can an algorithm know the “Real You”?: understanding people's reactions to hyper-personal analytics systems, in Proceedings of the 33rd Annual ACM Conference on Human Factors in Computing Systems (CHI '15) (New York, NY:ACM), 797–806. 10.1145/2702123.2702274

